# Inflammatory mediators in breast cancer: Coordinated expression of TNFα & IL-1β with CCL2 & CCL5 and effects on epithelial-to-mesenchymal transition

**DOI:** 10.1186/1471-2407-11-130

**Published:** 2011-04-12

**Authors:** Gali Soria, Maya Ofri-Shahak, Ilana Haas, Neora Yaal-Hahoshen, Leonor Leider-Trejo, Tal Leibovich-Rivkin, Polina Weitzenfeld, Tsipi Meshel, Esther Shabtai, Mordechai Gutman, Adit Ben-Baruch

**Affiliations:** 1 Department of Cell Research and Immunology, George S. Wise Faculty of Life Sciences, Tel Aviv University, Israel; 2 Department of Surgery A, Meir Medical Center and the Sackler School of Medicine, Tel Aviv University, Israel; 3 Department of Oncology, Tel Aviv Sourasky Medical Center, and the Sackler School of Medicine, Tel Aviv University, Israel; 4 Department of Pathology, Tel Aviv Sourasky Medical Center, and the Sackler School of Medicine, Tel Aviv University, Israel; 5 Statistical Service, Tel Aviv Sourasky Medical Center, and the Sackler School of Medicine, Tel Aviv University, Israel; 6 Department of Surgery B, Sheba Medical Center and the Sackler School of Medicine, Tel Aviv University, Israel

## Abstract

**Background:**

The inflammatory chemokines CCL2 (MCP-1) & CCL5 (RANTES) and the inflammatory cytokines TNFα & IL-1β were shown to contribute to breast cancer development and metastasis. In this study, we wished to determine whether there are associations between these factors along stages of breast cancer progression, and to identify the possible implications of these factors to disease course.

**Methods:**

The expression of CCL2, CCL5, TNFα and IL-1β was determined by immunohistochemistry in patients diagnosed with: (1) Benign breast disorders (=healthy individuals); (2) Ductal Carcinoma *In Situ *(DCIS); (3) Invasive Ducal Carcinoma without relapse (IDC-no-relapse); (4) IDC-with-relapse. Based on the results obtained, breast tumor cells were stimulated by the inflammatory cytokines, and epithelial-to-mesenchymal transition (EMT) was determined by flow cytometry, confocal analyses and adhesion, migration and invasion experiments.

**Results:**

CCL2, CCL5, TNFα and IL-1β were expressed at very low incidence in normal breast epithelial cells, but their incidence was significantly elevated in tumor cells of the three groups of cancer patients. Significant associations were found between CCL2 & CCL5 and TNFα & IL-1β in the tumor cells in DCIS and IDC-no-relapse patients. In the IDC-with-relapse group, the expression of CCL2 & CCL5 was accompanied by further elevated incidence of TNFα & IL-1β expression. These results suggest progression-related roles for TNFα and IL-1β in breast cancer, as indeed indicated by the following: (1) Tumors of the IDC-with-relapse group had significantly higher persistence of TNFα and IL-1β compared to tumors of DCIS or IDC-no-relapse; (2) Continuous stimulation of the tumor cells by TNFα (and to some extent IL-1β) has led to EMT in the tumor cells; (3) Combined analyses with relevant clinical parameters suggested that IL-1β acts jointly with other pro-malignancy factors to promote disease relapse.

**Conclusions:**

Our findings suggest that the coordinated expression of CCL2 & CCL5 and TNFα & IL-1β may be important for disease course, and that TNFα & IL-1β may promote disease relapse. Further *in vitro *and *in vivo *studies are needed for determination of the joint powers of the four factors in breast cancer, as well as analyses of their combined targeting in breast cancer.

## Background

Breast cancer provides a typical example of an inflammation-linked malignant disease. Breast tumors are enriched with inflammatory constituents, including cells that are polarized to the tumor-promoting phenotype, and soluble factors. Cumulative findings of a large number of studies indicate that many of the inflammatory components present in the tumor microenvironment actively support breast cancer development and progression [1-4].

Recently, much attention has been given to the roles of inflammatory chemokines and cytokines in breast cancer, with emphasis on the chemokines CCL2 and CCL5, and the cytokines tumor necrosis factor α (TNFα) and interleukin 1β (IL-1β). While each component has been studied meticulously, too little consideration has been given to possible associations and interactions between the inflammatory mediators, and to their joint presence, as part of the inflammatory microenvironment, in breast cancer. The identification of the inflammatory setup prevailing in breast cancer may be instrumental in providing us with a better basis for the future design of improved therapeutic modalities, and of advanced diagnostic and prognostic tools.

In this study, we asked if associations exist between the inflammatory chemokines CCL2 & CCL5 and the inflammatory cytokines TNFα & IL-1β in breast cancer. All four factors are expressed in breast tumors, they exert a diverse array of activities that support malignancy and they were shown to be directly involved in promoting tumor growth and metastasis in animal model systems of breast cancer (Refs [3,5-24] for CCL2 & CCL5, [25-59] for TNFα and IL-1β). Out of the four factors, TNFα is of special interest because of reports showing that under specific circumstances it may have cytotoxic and anti-tumor effects in several malignant diseases [25,60,61].

The chemokines CCL2 and CCL5 are categorized as "inflammatory chemokines", and as such they are usually not constitutively expressed by normal tissue cells. Rather, their expression is induced by inflammatory insults that prevail in the course of immune functions, including by stimulation with TNFα and IL-1β. The inter-connection between CCL2 & CCL5 and TNFα & IL-1β in the immune setting suggests that similar interactions exist between these chemokines and cytokines also within the inflammatory context of breast tumors. It is possible that TNFα & IL-1β and the two chemokines are interrelated in breast tumor cells, and that these four factors establish associations that may eventually contribute to tumor growth and metastasis.

To determine if this indeed the case, we have initiated this study by determining the expression patterns of CCL2, CCL5, TNFα and IL-1β in biopsy sections of healthy individuals and in breast cancer patients at different progression stages of disease. While only negligible staining of the factors was detected in infiltrating leukocytes in all groups, there was substantial expression of all four factors in breast tumor cells. Therefore, we focused in this study on the identification of expression patterns of CCL2, CCL5, TNFα and IL-1β in normal and malignant breast epithelial cells, and their potential contribution to disease course.

The results of our study indicate that significant associations exist between CCL2 & CCL5 and TNFα & IL-1β in breast cancer, along different stages of disease course. Furthermore, the results of this study support the possibility that TNFα & IL-1β play an important role in disease progression. These two cytokines were found to be highly persistent in tumors of patients suffering from relapsed disease. In addition, we found that they promoted processes that are required for disease progression and local recurrence, such as EMT which was induced mainly by TNFα.

The tumor-promoting activities of the inflammatory mediators CCL2 & CCL5 and TNFα & IL-1β in breast cancer are not fully overlapping (references above), therefore their coordinated expression may eventually provide advantage to the developing and metastasizing tumor. This possibility emphasizes the need to determine the joint powers of the four factors together in breast cancer, by using in vitro and in vivo model systems. Moreover, our results suggest an important role for TNFα in breast malignancy, because the cytokine has substantial ability to promote progression-related processes by inducing EMT processes in the tumor cells.

## Methods

### Patients

This study included 38 healthy individuals who were diagnosed with benign breast disorders that included fibrocystic changes, hyperplastic changes and benign tumors (this group is termed herein "Benign"). The study also included 88 breast cancer patients, who were divided to three groups: (1) 30 patients with DCIS. Since DCIS is a less malignant form of disease that rarely develops to IDC [62-64], the DCIS group included patients in whom disease has not progressed to IDC in the course of 5-10 years of follow up; (2) 23 patients with IDC, who remained disease free in the course of 5-10 years of follow up (IDC-no-relapse); (3) 35 patients with IDC who relapsed with metastases or local tumor/s, or who died of breast cancer, in 5-10 years of follow up (IDC-with-relapse).

The characteristics of the study patients who were diagnosed with DCIS, IDC-no-relapse and IDC-with-relapse are presented in Table 1. Tumor status (T1, etc.) was determined by the guidelines of the Cancer Staging Manual of the American Joint Committee on Cancer. The patients were treated and followed in Tel Aviv Sourasky Medical Center and in Meir Medical Center. The study was approved by the Helsinki Committees of both Centers as a retrospective study based on information obtained from files of unidentified patients (included in the category of studies which do not require informed consent from study patients).

**Table 1:** The characteristics of the DCIS, IDC-no-relapse and IDC-with-relapse patients included in the study

Patient characteristics		DCIS		IDC - No Relapse		IDC - With Relapse	
		n = 30	(%)	n = 23	(%)	n = 35	(%)
Menopause status	Pre-menopause	3	(10.0%)	0	(0.0%)	7	(20.0%)
	Menopause	7	(23.3%)	0	(0.0%)	0	(0.0%)
	Post-menopause	8	(26.7%)	0	(0.0%)	25	(71.4%)
	Unknown	12	(40.0%)	23	(100.0%)	3	(8.6%)

Node status	N0	30	(100.0%)	14	(60.9%)	17	(48.6%)
	N1	0	(0.0%)	4	(17.4%)	14	(40.0%)
	N2	0	(0.0%)	1	(4.4%)	0	(0.0%)
	Unknown	0	(0.0%)	4	(17.4%)	4	(11.4%)

Tumor	T0	26	(86.7%)	0	(0.0%)	1	(2.9%)
	T1	0	(0.0%)	2	(8.7%)	14	(40.0%)
	T≥2	0	(0.0%)	19	(82.6%)	17	(48.6%)
	Unknown	4	(13.3%)	2	(8.7%)	3	(8.5%)

Grade	1	5	(16.7%)	6	(26.1%)	5	(14.3%)
	2	12	(40.0%)	9	(39.1%)	10	(28.6%)
	3	6	(20.0%)	5	(21.7%)	7	(20.0%)
	Unknown	7	(23.3%)	3	(13.1%)	13	(37.1%)

Radiotherapy	Yes	19	(63.4%)	17	(73.9%)	28	(80.0%)
	No	10	(33.3%)	5	(21.7%)	5	(14.3%)
	Unknown	1	(3.3%)	1	(4.4%)	2	(5.7%)

Chemotherapy	Yes	0	(0.0%)	17	(73.9%)	15	(42.9%)
	No	18	(60.0%)	5	(21.7%)	17	(48.6%)
	Unknown	12	(40.0%)	1	(4.3%)	3	(8.5%)

Her2-neu	Yes	3	(10.0%)	1	(4.3%)	6	(17.1%)
	No	1	(3.3%)	15	(65.3%)	26	(74.3%)
	Unknown	26	(86.7%)	7	(30.4%)	3	(8.6%)

ER	Yes	23	(76.7%)	20	(87.0%)	23	(65.7%)
	No	2	(6.7%)	2	(8.7%)	10	(28.6%)
	Unknown	5	(16.6%)	1	(4.3%)	2	(5.7%)

PR	Yes	17	(56.7%)	19	(82.6%)	19	(54.3%)
	No	7	(23.3%)	3	(3.0%)	13	(37.1%)
	Unknown	6	(20.0%)	1	(4.4%)	3	(8.6%)

### Immunohistochemistry

In this study, we have used archived paraffin blocks of patients, all obtained at the time of diagnosis. Serial sections (5 μm thick) were prepared from the blocks and processed for immunohistochemistry (IHC) by the Pathology Departments of Tel Aviv Sourasky Medical Center and Meir Medical Center.

The biopsy sections were deparaffinized, dehydrated in xylene and graded alcohols, rinsed in PBS and stained by primary antibodies. All antibodies used in IHC were commercial monoclonal antibodies, and their ability to recognize their respective antigens was confirmed in ELISA assays (data not shown). Staining by primary monoclonal antibodies for human CCL5 (PeproTech, Rocky Hill, NJ; Cat# 500-M75) and to human CCL2 (R&D Systems, Minneapolis, MN; Cat# MAB679) was performed by two protocols that showed similar sensitivity and specificity: (1) Sections were incubated with hyaluronidase at 37°C for 1 hr, then treated with 3% H_2_O_2 _for 10 min at room temperature (RT). After rinsing in PBS, non-specific binding was blocked by incubating the sections with normal goat serum at 37°C for 30 min. Then, the sections were stained over night with the primary antibodies at 4°C, except for sections of DCIS patients in which the staining was performed for 4 hr (the time was reduced in this case due to some background staining that was observed only in the DCIS biopsies stained by the different antibodies). (2) Sections were immersed in a staining dish containing citrate buffer at 95-100°C for 22 min, then cooled for 20 min. After washing with PBS, the sections were blocked with blocking solution (0.05% sodium azide and 0.05% Tween 20 in PBS). Next, the sections were incubated with the different primary antibodies for 1 hr at RT, blocked by 3% H_2_O_2 _for 10 min at RT, and washed thoroughly in PBS.

Staining for TNFα and IL-1β was performed according to the second procedure detailed above, using monoclonal antibodies to human TNFα (PeproTech; Cat# 500-M26) and to human IL-1β (Exalpha, Watertown, MA; Cat# L140M).

For all four factors (CCL2, CCL5, TNFα and IL-1β), the staining by secondary antibodies was performed as follows: sections were washed thoroughly in PBS, stained with biotinylated anti broad spectrum secondary antibody, or alternatively, by anti mouse secondary antibody (both having similar sensitivity and specificity) for 10 min at RT. After additional washing in PBS, the sections were stained by HRP-streptavidin for 10 min at RT. Then, the sections underwent additional washings in PBS and were incubated with Diaminobenzidine for 10 min at RT, washed and counterstained by incubation with hematoxylin for 5 sec at RT. After washing in H_2_O, the sections were dehydrated in graded alcohols and mounted to cover slides.

In all cases, negative controls included substitution of the primary antibodies by PBS, or by non-relevant isotype controls. The specificity of the antibodies against TNFα and IL-1β was verified using mouse IgG1 as an isotype control for the antibodies (that shared the same isotype). Also, since the antibodies against CCL5 share the same isotype with the antibodies against CCL2, but there were biopsies that stained with one but not with the other, they served as internal controls for each other's specificity (In the 88 breast cancer patients: 9% of the biopsies stained for CCL2 but not for CCL5, and 25% of the biopsies stained for CCL5 but not for CCL2; In all other cases, the expression of CCL2 was coordinated with that of CCL5).

All slides were submitted to light microscopy, and the staining pattern in the cells was evaluated in a blind manner by a pathologist having expertise in breast cancer, and by a pathology-experienced researcher. The tumors were evaluated for % chemokine/cytokine-positive cells in the tumors (0-100%) and intensity of expression of the chemokines/cytokines (1 = low intensity, 2 = medium intensity, 3 = high intensity). TNFα and IL-1β were also evaluated for score of expression, determined as "% cytokine-positive cells in the tumors × the intensity of cytokine expression in each of the tumors". In line with the evaluations of many other markers in IHC, a threshold for positive staining was set. Based on our past experience, non-specific staining (exhibited as rare/sporadic/random stainings) could account for up to 5% of the epithelial cells in the biopsy, therefore the cut-off for positivity was set on 5%.

The data on expression of Her2-neu, estrogen receptor α (ER) and progesterone receptor (PR) that are included in Table 1 were provided by Tel Aviv Sourasky Medical Center and by Meir Medical Center, where the patients were treated and followed.

### Statistical analyses of immunohistochemistry

In this study, a comparison was made between the incidences of expression the different factors in normal breast epithelial duct cells of the Benign group and the tumor cells in DCIS, IDC-no-relapse and IDC-with-relapse groups (Fig. two). This analysis was performed by Chi-square or two-tailed Fisher's exact test as applicable. Hochberg's GT2 method for multiple comparisons was applied for pair-wise comparisons between groups of patients.

The differences between the incidences of expression of the different factors in normal breast epithelial cells that were adjacent to tumor cells in the cancer biopsies, as compared to the tumor cells in the DCIS, IDC-no-relapse and IDC-with-relapse groups (Table 2), were determined by McNemar test (except for TNFα expression in "IDC-no-relapse" group, that because of limitations of the McNemar test was analyzed by Wilcoxon).

Chi-square or Fisher's exact test were used in order to determine the significance of associations between factors that belonged to "Group 1" (TNFα & IL-1β) and factors that belonged to "Group 2" (CCL2 & CCL5) (Fig. three).

Group means were compared using a one-way Analysis of Variance (Fig. seven and Table 3). Whenever a significant effect of group was observed, pair-wise comparisons between groups were performed using the Ryan-Einot-Gabriel-Welsch Multiple Range Test. Levene's Test for Homogeneity of Variances was applied to compare dispersion between groups.

Logistic regression model was applied to assess the potential use of Her2-neu, ER and PR with cytokines as risk factors for disease relapse.

All statistical analyses were performed using the SAS for Windows version 9.1.3.

### Determination of EMT properties

The T47D and MCF-7 human breast carcinoma cell lines were grown in DMEM medium as described previously [20]. The growth medium was replaced by serum-deficient medium, and the cells were grown to confluency in the absence or in the presence of TNFα or IL-1β for 72 hr.

E-cadherin expression was determined in live cells by mouse antibodies against human E-cadherin (Santa Cruz Biotechnology, Santa Cruz, CA), followed by FITC-conjugated antibodies against mouse IgG (Jackson ImmunoResearch Laboratories, West Grove, Pennsylvania). Baseline staining was obtained by adding the appropriate buffer to the cells instead of primary antibody, and by staining with non-relevant isotype-treated antibodies. Determination of vimentin expression was performed in methanol-treated cells by mouse antibodies against human vimentin (Santa Cruz Biotechnology), followed by FITC-conjugated antibodies against mouse IgG (Jackson ImmunoResearch Laboratories). Baseline staining was obtained by non-relevant isotype-matched antibodies. Stainings were determined with a Becton Dickinson FACSort (Mountain View, CA) using the CellQuest software.

The expression of β-catenin was determined by specific antibodies, along with nuclei staining by DAPI. The cells were fixed by 8% paraformaldehyde for 15 min. Following treatment by 0.2% triton for 10 min, blocking was performed with PBS containing 2% BSA for 30 min. Primary mouse antibodies to human β-catenin were then used (Santa Cruz Biotechnologies), followed by FITC-conjugated mouse antibodies against mouse IgG. Baseline staining was obtained by adding the appropriate buffer to the cells instead of primary antibody. Similarly fixed and permeabilized cells were incubated with phalloidin-Alexa 488 (Molecular Probes, PoortGebouw, The Netherlands) for detection of actin organization. Stained cells were detected by LSM 510 Meta confocal microscope (Carl Zeiss AG, Oberkochen, Germany).

In adhesion assays, TNFα-stimulated cells were trypsinized, and then plated in 96 well non-tissue culture plates for 2 hr at 37°C. Non-adherent cells were removed by washing in PBS. Detection of adherent cells was performed by an alkaline phosphatase assay, by the addition of alkaline phosphatase substrate buffer, containing 3 mg/ml p-nitrophenyl phosphate disodium (Sigma-Aldrich, St. Louis, MO). Following the addition of 1M NaOH, optical density was measured at 405 nm. Statistical analysis was performed by Student's *t *test.

Migration and invasion assays were performed in transwell migration chambers with 8-μm pore size (Costar, Cambridge, MA). In both assays, MCF-7 cells were stimulated by TNFα for 48 hr, then added to the upper wells of the chamber with or without TNFα (in serum-free DMEM). In invasion assays, the upper wells were pre-coated with matrigel (20 μg/ml in serum-free cold DMEM; BD Biosciences, Bedford, MA) for 1 hr at 37°C, then blocking was performed for additional 1 hr with 0.1% heat inactivated BSA (diluted in PBS). In both assays, the lower wells of the chamber included 500 μl of DMEM supplemented with 10% FCS. Following 21-23 hr of incubation, the cells on the upper surface of the filter were completely removed by wiping with a cotton swab. The filters were fixed (by methanol in migration assays and by 4% paraformaldehyde in invasion assays) and were stained with Diff-Quik (Dade Behring, Newark, DE). The cells were counted in high power fields (HPF) by light microscopy. Statistical analysis was performed by Student's *t *test.

## Results

### Patterns of expression of inflammatory chemokines and cytokines, determined by immunohistochemistry

**Figure 1:**
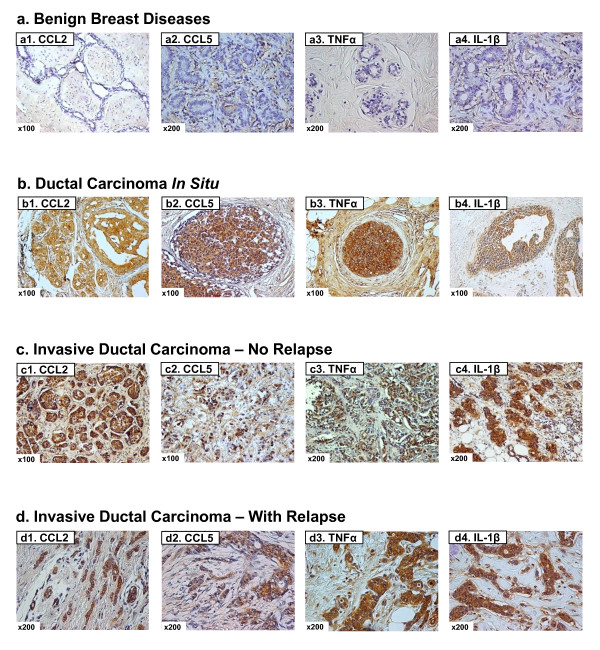
**Expression patterns of CCL2, CCL5, TNFα and IL-1β in healthy individuals and breast cancer patients**. Representative examples of the expression of CCL2, CCL5, TNFα and IL-1β in the different groups of patients included in the study, in biopsies obtained at the time of diagnosis. **(a1-a4) **Patients diagnosed with benign breast disorders. The pictures demonstrate the lack of staining of the four factors in the normal breast epithelial cells, as denoted in the majority of patients included in this group. **(b1-b4) **DCIS patients. The pictures demonstrate positive staining of the four factors in the malignant lesions, as denoted in the majority of patients included in this group. **(c1-c4) **IDC-no-relapse patients. The pictures demonstrate positive staining of the four factors in the tumor cells, as denoted in the majority of patients included in this group. **(d1-d4) **IDC-with-relapse patients. The pictures demonstrate positive staining of the four factors in the tumor cells, as denoted in the majority of patients included in this group. (a1, b1, c1, d1) CCL2 staining; (a2, b2, c2, d2) CCL5 staining; (a3, b3, c3, d3) TNFα staining; (a4, b4, c4, d4) IL-1β staining. The expression of the proteins was determined by IHC using specific antibodies, whose specificity in IHC was verified. The values of photo magnification are indicated in the left bottom corner of each of the pictures.

Biopsy sections were obtained from the total of 126 individuals (as indicated in "Materials and methods") at the time of diagnosis, and were stained by specific antibodies that recognized human CCL2, CCL5, TNFα or IL-1β. Figure 1 displays representative examples of the staining patterns of the four proteins in biopsy sections of healthy individuals (the "Benign" group), and of DCIS, IDC-no-relapse and IDC-with-relapse patients. While the expression of CCL2, CCL5, TNFα or IL-1β was only minimally detected in infiltrating leukocytes of all groups of patients, the four factors were clearly observed in breast tumor cells, with mainly a cytoplasmic staining pattern. Based on the above, we have performed detailed analysis of the expression patterns of the four factors in breast epithelial cells, normal and malignant, in biopsy sections of healthy individuals and of breast cancer patients, as indicated below.

### Protein expression in normal and malignant epithelial breast cells

**Figure 2:**
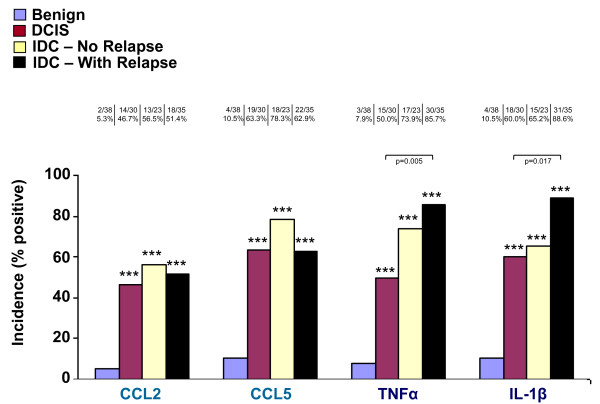
**Incidence of expression of CCL2, CCL5, TNFα and IL-1β in healthy individuals and breast cancer patients**. The expression of CCL2, CCL5, TNFα and IL-1β was determined by IHC in four groups of patients, in biopsies obtained at the time of diagnosis: Benign (n = 38), DCIS (n = 30), IDC-no-relapse (n = 23) and IDC-with-relapse (n = 35). The incidences of expression of the four factors are diagrammed according to the type of factor analyzed. The number of patients positive for protein expression is indicated above the relevant bar, and the incidence is presented in percentages. ***p < 0.001 for the differences between the incidences of expression of each factor in the tumor cells of DCIS/IDC-no-relapse/IDC-with-relapse, and the incidence of its expression in the normal cells of the Benign group. No significant differences were denoted with respect to CCL2 and CCL5 expression in the tumor cells between the DCIS, IDC-no-relapse and IDC-with-relapse groups. For TNFα and IL-1β expression in the tumor cells, significant elevations were denoted only for the incidence of expression between the DCIS and the IDC-with-relapse groups (TNFα: p = 0.005; IL-1β: p = 0.017).

Figure 2 provides information on the incidences of expression of CCL2, CCL5, TNFα and IL-1β in normal breast epithelial cells of Benign patients, and in the tumor cells in patients diagnosed with DCIS, IDC-no-relapse and IDC-with-relapse. All four factors were minimally detected in normal breast epithelial duct cells in the healthy individuals of the Benign group (expression incidences ranging between 5.3% and 10.5%). A significant elevation was denoted in the expression of CCL2, CCL5, TNFα and IL-1β in the tumor cells in all groups of cancer patients (DCIS, IDC-no-relapse and IDC-with-relapse), when compared to their expression in the normal breast cells in biopsies of the Benign patients, with significance value of p < 0.001 in all cases.

In parallel, we determined the expression of CCL2, CCL5, TNFα and IL-1β in normal breast epithelial duct cells that were in proximity to the tumor cells in biopsies of patients with DCIS, IDC-no-relapse and IDC-with-relapse (Table 2). In these three groups of cancer patients, we found low incidence of expression of the inflammatory chemokines and inflammatory cytokines in the normal cells adjacent to the tumor cells, while they were expressed at significantly higher incidence in the tumor cells (incidences and p values are presented in Table 2). The incidence of CCL2, CCL5, TNFα and IL-1β expression in the normal epithelium was not significantly different between DCIS and IDC-no-relapse, and also did not differ significantly between DCIS and IDC-with-relapse patients.

**Table 2:** The incidence of expression of CCL2, CCL5, TNFα and IL-1β in tumor cells and in adjacent normal breast epithelial cells in DCIS and IDC patients

Expression of chemokines/cytokines in	Number of patients (%)			
	
	With CCL2 expression	With CCL5 expression	With TNFα expression	With IL-1β expression
**DCIS**				

**Breast malignant cells**	11/27 (40.7%)	16/27 (59.2%)	13/27 (48.1%)	15/27 (55.5%)

**Adjacent normal breast epithelial duct cells**	5/27 (18.5%)	7/27 (25.9%)	3/27 (11.1%)	6/27 (22.2%)

**p value**	0.03	0.01	0.002	0.003

**IDC-no-relapse**				

**Breast malignant cells**	10/19 (52.6%)	15/19 (78.9%)	14/19 (73.7%)	13/19 (68.4%)

**Adjacent normal breast epithelial duct cells**	1/19 (5.3%)	1/19 (5.3%)	0/19 (0.0%)	1/19 (5.3%)

**p value**	0.003	0.0002	0.0001	0.0005

**IDC-with-relapse**				

**Breast malignant cells**	15/29 (51.7%)	17/29 (58.6%)	24/29 (82.7%)	25/29 (86.2%)

**Adjacent normal breast epithelial duct cells**	3/29 (10.3%)	2/29 (6.9%)	6/29 (20.7%)	8/29 (27.6%)

**p value**	0.001	0.0001	<0.0001	<0.0001

The expression CCL2, CCL5, TNFα and IL-1β was determined by IHC in serial biopsy sections of primary tumors of the breast taken at the time of diagnosis, from patients diagnosed with DCIS, IDC-no-relapse, or IDC-with-relapse. In this analysis, the incidences of expression of the four factors were determined in the malignant cells and in adjacent normal breast epithelial cells, in the same biopsy. Therefore, this analysis included only patients in whom normal ducts and breast tumor cells were detected in the same biopsy: DCIS - n = 27; IDC-no-relapse - n = 19; "IDC-with-relapse - n = 29. The incidence of CCL2, CCL5, TNFα and IL-1β expression in the normal epithelium was not significantly different between DCIS and IDC-no-relapse, and also did not differ significantly between DCIS and IDC-with-relapse patients. The expression of the proteins was determined by antibodies whose binding specificity in IHC was verified.

Taken together, these findings indicate that the expression of the inflammatory chemokines and inflammatory cytokines was acquired by breast epithelial cells upon their malignant transformation.

### Protein expression along stages of disease progression

Next, we compared the three groups of breast cancer patients (DCIS, IDC-no-relapse, IDC-with-relapse) with respect to the incidence of expression of CCL2, CCL5, TNFα and IL-1β in the tumor cells. The results of Figure 2 clearly show that the expression of the inflammatory chemokines CCL2 and CCL5 remained high and constant in the tumor cells of DCIS, IDC-no-relapse and IDC-with-relapse patients. No significant differences were denoted between DCIS, IDC-no-relapse and IDC-with-relapse patients, with respect to the incidence of CCL2 and CCL5 expression in the tumor cells.

A high incidence of expression in the tumor cells was also denoted for TNFα and IL-1β in the three groups of cancer patients: DCIS, IDC-no-relapse and IDC-with-relapse. The results of Figure 2 indicate that the incidence of TNFα expression was 50% in DCIS patients and was significantly increased to 85.7% in IDC-with-relapse patients (p = 0.005); Similarly, the incidence of IL-1β was significantly raised from 60% in DCIS to 88.6% in IDC-with-relapse patients (p = 0.017). No other significant differences were found between the different groups of breast cancer patients with respect to TNFα and IL-1β expression in the tumor cells (namely: IDC-no-relapse *vs*. DCIS; IDC-no-relapse *vs*. IDC-with-relapse).

Together, these findings indicate that there was high incidence of CCL2, CCL5, TNFα and IL-1β expression in the tumor cells in the three groups of cancer patients, and that the expression of TNFα and IL-1β was further elevated in the IDC-with-relapse group. The rare expression of all four factors in normal breast epithelial cells, and the high incidence of their expression in tumor cells in biopsies of all groups of cancer patients, indicate that the expression of the inflammatory chemokines CCL2 & CCL5 is coordinated with that of the inflammatory cytokines TNFα & IL-1β along stages of tumor development and progression in breast cancer.

### Dependence between the inflammatory chemokines and the inflammatory cytokines along disease progression

Based on the above results, we asked whether there are associations between the inflammatory chemokines CCL2 & CCL5 and the inflammatory cytokines TNFα & IL-1β in patients representing different stages along the progression process of breast cancer. To this end, we divided the four factors to two groups, based on their published functional interactions in breast tumor cells: Group 1 - The inflammatory cytokines TNFα & IL-1β, which promote the release of CCL2 & CCL5 by breast tumor cells; Group 2 - The inflammatory chemokines CCL2 & CCL5, whose release by malignant breast cells is increased by TNFα and IL-1β (Please refer to Additional Files 1 and 2, as well as to references [65-70]. These analyses were performed in the tumor cells in the three groups of cancer patients: DCIS, IDC-no-relapse and IDC-with-relapse.

**Figure 3:**
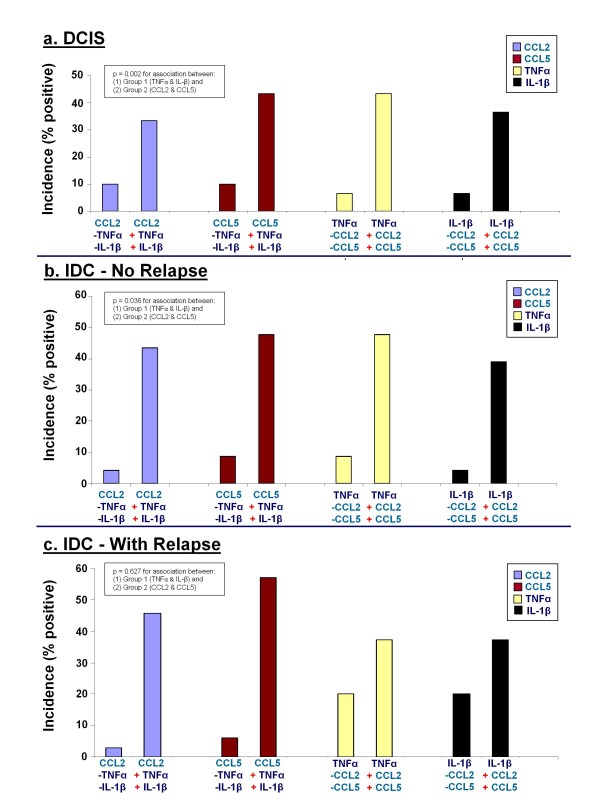
**The associations between the inflammatory chemokines CCL2 & CCL5 and the inflammatory cytokines TNFα & IL-1β, in breast cancer patients**. The analyzed factors were sub-divided to two groups: Group 1 - The inflammatory cytokines TNFα & IL-1β; Group 2 - The inflammatory chemokines CCL2 & CCL5; Detailed explanation of graphs A, B and C is provided further below. **(a) **In DCIS patients, p = 0.002 for associations between Group 1 and Group 2. **(b) **In IDC-no-relapse patients, p = 0.036 for associations between Group 1 and Group 2. **(c) **In IDC-with-relapse patients, p = 0.627 for associations between Group 1 and Group 2. Explanation: In each group of breast cancer patients (DCIS, IDC-no-relapse, IDC-with-relapse), the graphs show the following: (1) CCL2: The graph shows the incidence of CCL2 expression when it was co-expressed with TNFα and IL-1β in the same biopsy [presented in the graph as "CCL2 (+ TNFα) (+ IL-1β)"], as compared to the incidence of CCL2 expression when it was NOT co-expressed with TNFα and IL-1β in the same biopsy [presented in the graph as "CCL2 (- TNFα) (- IL-1β)"]; (2) CCL5: The graph shows the incidence of CCL5 expression when it was co-expressed with TNFα and IL-1β in the same biopsy [presented in the graph as "CCL5 (+ TNFα) (+ IL-1β)"], as compared to the incidence of CCL5 expression when it was NOT co-expressed with TNFα and IL-1β in the same biopsy [presented in the graph as "CCL5 (- TNFα) (- IL-1β)"]; (3) TNFα: The graph shows the incidence of TNFα expression when it was co-expressed with CCL2 and CCL5 in the same biopsy [presented in the graph as "TNFα (+ CCL2) (+ CCL5)"], as compared to the incidence of TNFα expression when it was NOT co-expressed with CCL2 and CCL5 in the same biopsy [presented in the graph as "TNFα (- CCL2) (- CCL5)"]; (4) IL-1β: The graph shows the incidence of IL-1β expression when it was co-expressed with CCL2 and CCL5 in the same biopsy [presented in the graph as "IL-1β (+ CCL2) (+ CCL5)"], as compared to the incidence of IL-1β expression when it was NOT co-expressed with CCL2 and CCL5 in the same biopsy [presented in the graph as "IL-1β (- CCL2) (- CCL5)"];

When we analyzed the DCIS group, we found that factors belonging to Group 1 (TNFα & IL-1β) "tended" to be expressed in the same biopsies in which the factors of group 2 were expressed (CCL2 & CCL5) (Figure 3a). For example, the study of CCL2 showed that CCL2 was co-expressed with TNFα and IL-1β in 33.3% of the DCIS biopsies. In contrast, in only 10% of the DCIS patients CCL2 was detected without co-expression of TNFα and IL-1β. This finding indicates that there was a preferential expression of CCL2, as a representative of Group 2, with factors that belonged to Group 1, namely TNFα & IL-1β.

Similar analyses that were performed for the other three factors in DCIS patients - CCL5, TNFα and IL-1β - have shown similar trends to those obtained for CCL2, namely that the inflammatory chemokines "favored" co-expression with the inflammatory cytokines, and *vice versa*. Accordingly, the statistical analyses that were preformed in DCIS patients demonstrated that the factors of Group 1 were significantly associated with the factors of Group 2 (p = 0.002), indicating that in this group of patients there were defined associations between the inflammatory chemokines and the inflammatory cytokines.

Thereafter, analyses that were performed for patients diagnosed with IDC-no-relapse have shown that the expression of CCL2 & CCL5 was coordinated with that of TNFα & IL-1β (Figure 3b). This was substantiated by significant associations that were detected between factors of Group 1 (TNFα & IL-1β) and Group 2 (CCL2 & CCL5), with p = 0.036.

A somewhat different pattern of expression was obtained for IDC-with-relapse patients (Figure 3c). As in the DCIS and IDC-no-relapse groups, the incidence of CCL2 and CCL5 in the IDC-with-relapse group remained at the level of 50-65%; however, the incidence of TNFα and IL-1β expression in the IDC-with-relapse group was increased to 85-90% (Figure 2). Therefore, in many of the patients of this group, the expression of TNFα & IL-1β was coordinated with that of CCL2 & CCL5, but in others the expression of TNFα & IL-1β was not accompanied by expression of CCL2 & CCL5. This has led to insignificant associations (p = 0.627) between Group 1 (the inflammatory cytokines TNFα & IL-1β) and Group 2 (the inflammatory chemokines CCL2 & CCL5) in the IDC-with-relapse group.

To conclude, in patients that belonged to the DCIS and IDC-no-relapse groups, the expression of the inflammatory chemokines was coordinated and significantly associated with that of the inflammatory cytokines. In contrast, in the IDC-with-relapse group there was sub-population of patients in which the expression of CCL2 & CCL5 did not accompany the expression of TNFα & IL-1β. In this group of IDC-with-relapse patients, the expression of TNFα & IL-1β was further elevated and was highly prevalent, supporting an important role for TNFα and IL-1β in disease progression and recurrence. Furthermore, these results suggest that in this specific sub-group of IDC-with-relapse patients, the two cytokines are coordinated with other tumor-supporting factors that replace CCL2 & CCL5 in promoting disease relapse.

### The persistence of inflammatory cytokines in breast tumors may promote disease progression and recurrence, e.g. *via *epithelial-to-mesenchymal transition

The findings on the elevated expression of TNFα and IL-1β in the IDC-with-relapse group suggest that these two cytokines support disease progression. In addition to the tumor-promoting activities of TNFα and IL-1β that have already been described, it is possible that these two cytokines also have functional interactions with CCL2 and CCL5, by that promoting disease course. In line with this possibility, our findings have shown that TNFα and IL-1β up-regulated the release of CCL2 and CCL5 by breast tumor cells (Additional Files 1 and 2; also suggested by findings of references [65-70]). Such a cross-talk may lead to up-regulated expression of CCL2 and CCL5 in tumors expressing TNFα and/or IL-1β, thus possibly leading to increased tumor-supporting activities of the two chemokines in breast tumors (see Discussion, below).

In addition to their ability to promote the release of CCL2 and CCL5 by the tumor cells, it is possible that TNFα and IL-1β act directly to elevate processes that are required for local recurrence or metastasis formation, which identify the IDC-with-relapse group of patients. Recent studies indicate that acquisition of self-renewal properties that are required for formation of recurrent local tumors, as well as metastasis formation, are promoted by Epithelial-to-Mesenchymal Transition (EMT) processes [71-77]. Based on the above, we determined the possibility that TNFα and IL-1β promote EMT processes in the tumor cells, thus pushing forward disease recurrence and progression.

To investigate this possibility, we tested the ability of TNFα and IL-1β to induce in the tumor cells properties that are typical of EMT [71-77]. In these experiments, we used the T47D and MCF-7 cells, which represent a non-advanced stage of breast malignancy [78-82], and thus can serve as an appropriate platform for induction of EMT processes that are associated with more progressed disease.

**Figure 4:**
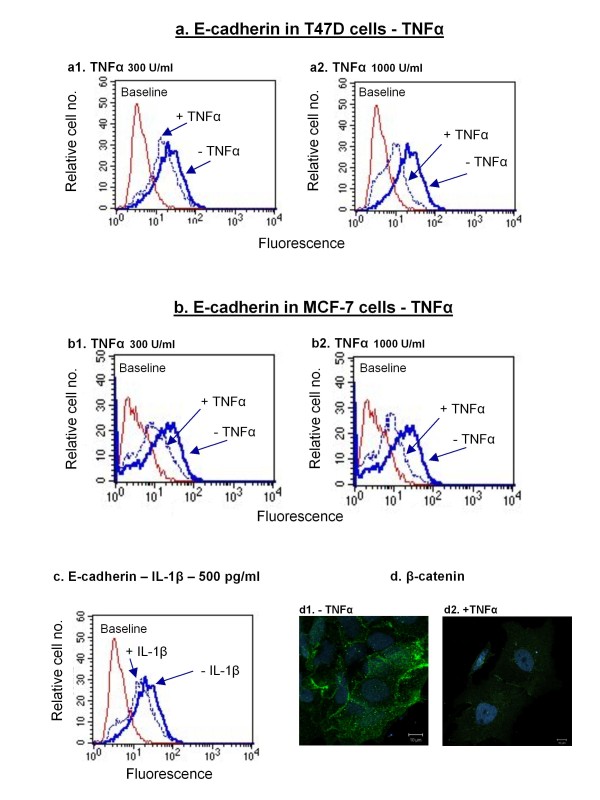
**Following stimulation by the inflammatory cytokines (mainly TNFα), the tumor cells show typical characteristics of EMT**. The T47D and MCF-7 human breast carcinoma cells were stimulated by TNFα and IL-1β for 72 hr. Note: The cytokine concentrations were chosen based on analyses in which their effects on the tumor cells were tested in a dose-dependent manner (data not shown). **(a) **Stimulation of T47D cells with 300 U/ml TNFα (a1) or with 1000 U/ml TNFα (a2). The membranous expression of E-cadherin was determined in live cells by flow cytometry. The figure presents the results of a representative experiment of n = 3, all showing similar results. **(b) **Stimulation of MCF-7 cells with 300 U/ml TNFα (b1) or 1000 U/ml TNFα (b2). The membranous expression of E-cadherin was determined in live cells by flow cytometry. The figure presents the results of a representative experiment of n > 3, all showing similar results. **(c) **T47D human breast carcinoma cells were stimulated by IL-1β (500 pg/ml). The membranous expression of E-cadherin was determined in live cells by flow cytometry. The figure presents the results of a representative experiment of n = 3, all showing similar results. **(d) **MCF-7 cells were stimulated by 1000 U/ml TNFα, and β-catenin expression (green) was determined by confocal analyses, in fixed cells. Nuclei are shown by blue DAPI staining. The figure presents the results of a representative experiment of n = 3, all showing similar results.

First, we stimulated the cells by TNFα and IL-1β (please see "Note" in legend to Figure 4 on cytokine concentrations used in these analyses), and determined the ability of the cytokines to induce reduction in the membranous expression of E-cadherin, an adhesion molecule which is important in establishing cell-to-cell contacts. The results of Figures 4a and 4b demonstrate that TNFα potently induced a very typical property of EMT, namely reduction in E-cadherin expression at the cell membrane of the tumor cells. This activity of TNFα was induced in both the T47D and the MCF-7 cells in a dose-dependent manner. In parallel, stimulation by IL-1β has also led to reduced expression of E-cadherin at the plasma membrane, but only in the T47D cells (Figure 4c).

**Figure 5:**
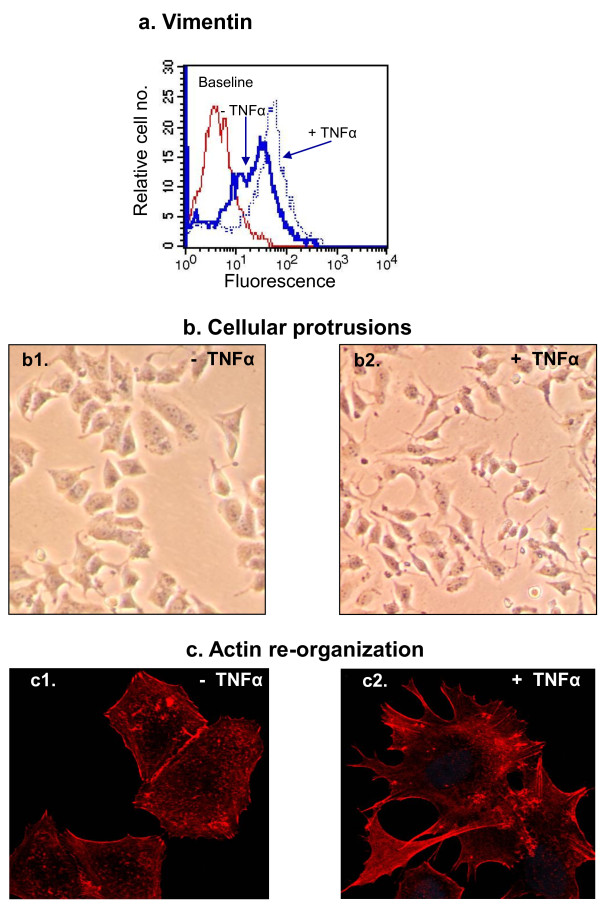
**Following stimulation by TNFα, breast tumor cells acquire a mesenchymal phenotype which is accompanied by protrusive characteristics**. MCF-7 breast carcinoma cells were stimulated by TNFα (1000 U/ml) for 72 hr. Thereafter, the following analyses were performed: **(a) **Vimentin expression, determined by specific antibodies in methanol-treated cells and analyzed by flow cytometry. The figure presents the results of a representative experiment of n = 3, all showing similar results. **(b) **Formation of cellular protrusions, determined by light microscopy. The figure presents the results of a representative experiment of n > 3, all showing similar results. **(c) **Actin polymerization, determined by phalloidin staining, analyzed by confocal microscopy. The figure presents the results of a representative experiment of n = 3, all showing similar results.

Based on these results and on additional preliminary findings (data not shown), we suspected that TNFα was a more potent inducer of EMT than IL-1β, and that its effects were more prominent on MCF-7 cells than on T47D cells. Therefore in depth analyses were continued with TNFα, analyzed on MCF-7 cells only. Further analyses indicated that TNFα has led to substantial decrease in the expression of β-catenin at the cell membrane of the tumor cells (Figure 4d), an event which is typical of EMT. Also, the stimulation by the cytokine has promoted the expression of vimentin, a mesenchymal feature whose expression is elevated in cells undergoing EMT (Figure 5a). In parallel, the tumor cells have acquired cellular protrusions, accompanied by extensive re-organization of the actin cytoskeleton (Figures 5b and 5c, respectively).

**Figure 6:**
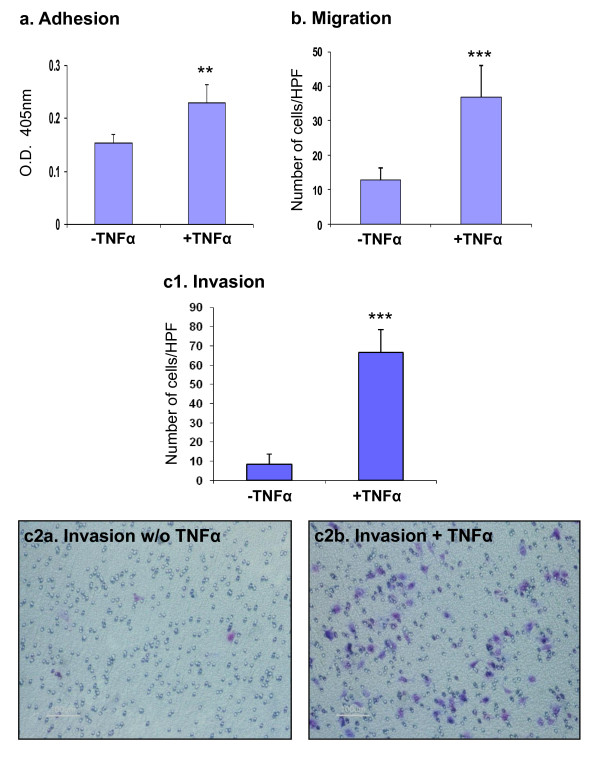
**Following stimulation by TNFα, breast tumor cells acquire increased adhesive, migratory and invasive properties**. MCF-7 breast carcinoma cells were stimulated by TNFα (1000 U/ml). Thereafter, the following analyses were performed: **(a) **Following 72 hr stimulation by TNFα, adhesion to substrate was determined by alkaline phosphatase assay. **p = 0.005 for the difference between cells that were stimulated or not stimulated by TNFα. **(b, c) **Determination of the ability of the cells to perform migratory and invasive activities, in response to serum. Following 48 hr stimulation by TNFα (1000 U/ml), migration and invasion assays were performed in transwells for 21-23 hr with or without TNFα stimulation. (b) Migration. ***p < 0.001 for TNFα-stimulated cells *vs*. non-stimulated cells. (c1) Invasion counts. ***p < 0.001 for TNFα-stimulated cells *vs*. non-stimulated cells. (c2) Invasion as demonstrated in light microscopy. HPF = High Power Field. In all parts of the figure, experiments are representatives of n = 3.

In addition to the above, TNFα has induced in the tumor cells elevated adhesion to substrate, a step necessary for establishment of productive metastases at remote organs (Figure 6a). Importantly, following TNFα stimulation, the cells have acquired the most critical and metastasis-relevant property of EMT, namely increased migratory and invasive properties (Figures 6b and 6c), by that substantiating the ability of the cytokine to induce EMT in breast tumor cells.

Taken together, the above results indicate that TNFα, and possibly also IL-1β - although to a lower extent - induced EMT properties in the tumor cells. Of note, similar activities of the two cytokines were obtained in an unrelated ongoing study in our laboratory, performed on a different set of breast cells (data not shown; manuscript in preparation), further substantiating the EMT-promoting activities of TNFα and IL-1β. Also, preliminary analyses that we have performed indicated that CCL2 and CCL5 were not capable of inducing EMT-related processes in the breast tumor cells (data not shown).

**Figure 7:**
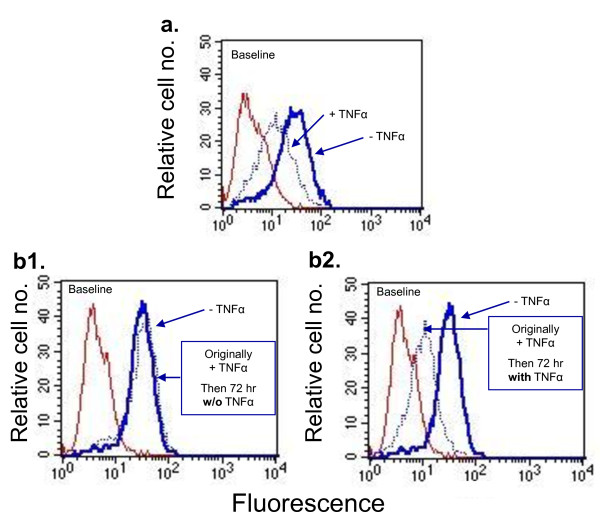
**In order to induce EMT, TNFα needs to be constantly present in vicinity to the tumor cells**. Determination of the reversibility of the TNFα-induced EMT effects. **(a) **MCF-7 cells were stimulated by TNFα (1000 U/ml) for 72 hr, followed by determination of membranous expression of E-cadherin in live cells, using flow cytometry. **(b) **The non-stimulated cells of part (A) were grown for additional 72 hr without TNFα. In contrast, the previously-stimulated cells (by TNFα) were grown for additional 72 hr without (b1) or with (b2) TNFα (1000 U/ml). Thereafter, the membranous expression of E-cadherin was determined in live cells by flow cytometry. The experiment is a representative of n = 3.

Since the potent EMT-promoting activities of TNFα may push forward processes of increased metastasis formation and disease recurrence, we further addressed the characteristics of TNFα activity. In view of reports showing that the EMT phenotype may be transient and needs continuous exposure to EMT-inducing factors [72], we asked if induction of EMT by TNFα required constant stimulation by the cytokine. To determine this issue, we have taken cells in which EMT was induced by TNFα (as indicated by reduced expression of E-cadherin at the cell membrane), and extended their growth for additional 72 hr with or without TNFα. The results of Figure 7 clearly indicate that the EMT phenotype was reversible if the cells were deprived of TNFα, while in cells that were grown continuously with the cytokine the EMT phenotype was preserved.

The above findings suggest that breast tumor cells benefit from high and constant presence of TNFα, because the persistence of the cytokine may induce EMT processes that lead to progressed and recurred disease. In such a case, we would expect that TNFα would be more persistent in tumors of IDC patients who relapsed with metastases or local tumors, compared to IDC-no-relapse or to DCIS patients.

**Figure 8:**
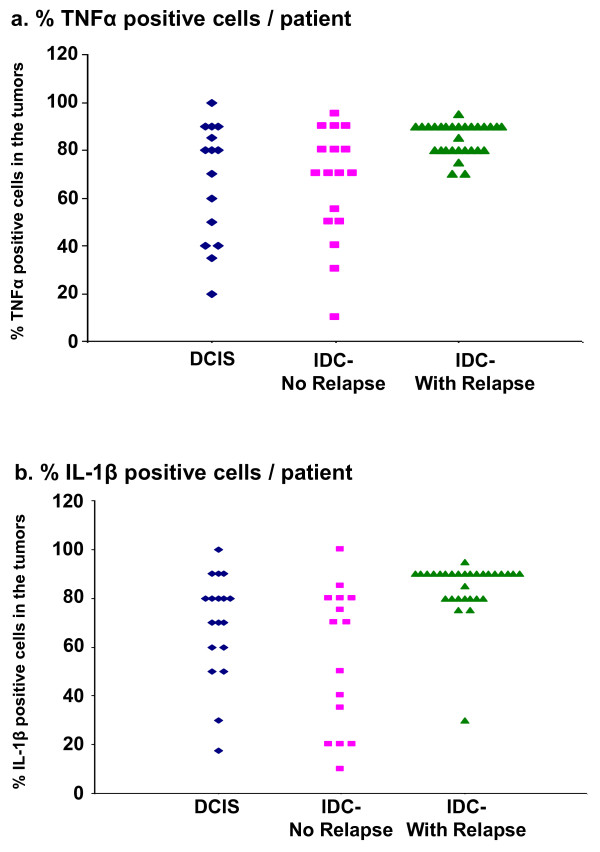
**There is high persistence of TNFα and IL-1β in tumors of IDC-with-relapse patients**. **(a) **Analyses of % cells that were positive for TNFα in tumors of breast cancer patients. The figure shows analyses that were performed only in patients whose tumors were positive for TNFα expression: DCIS (n = 15); IDC-no-relapse (n = 17); IDC-with-relapse (n = 29). Each symbol in the graph represents a single patient. Statistical values, as well as X ± SD are provided in Table 3. **(b) **Analyses of % cells that were positive for IL-1β in tumors of breast cancer patients. The figure shows analyses that were performed only in patients whose tumors were positive for IL-1β expression: DCIS (n = 18); IDC-no-relapse (n = 15); IDC-with-relapse (n = 31). Each symbol in the graph represents a single patient. Statistical values, as well as X ± SD are provided in Table 3.

To find out if this was indeed the case, we determined in each of the three groups of breast cancer patients (DCIS, IDC-no-relapse, IDC-with-relapse) the parameters of % TNFα-positive cells in the tumors, and the score of TNFα expression. The results presented in Figure 8a and in Table 3 demonstrate that there was a significant elevation in % TNFα-positive cells/patient in the IDC-with-relapse group when it was compared to DCIS and to the IDC-no-relapse groups, where p = 0.0008 for % TNFα-positive cells, and p = 0.0331 for TNFα score (as indicated in the legend to Table 3).

Of note, similar analyses that were performed for IL-1β have also shown highly significant persistence of this cytokine in tumors of IDC-with-relapse patients, with p < 0.0001 for % IL-1β-positive cells, and p = 0.0004 for IL-1β score (Figure 8b and Table 3). These results suggest that persistence of IL-1β in the tumors is also important for driving forward processes of progression and recurrence. Based on our data, these activities of IL-1β are not necessarily related to EMT, because this cytokine was not as effective in this function as TNFα. Rather, IL-1β may acquire activities that are related for example to elevated angiogenesis, as previously suggested [28,31,83]. In addition, it is possible that IL-1β acts jointly with other pro-malignancy factors to promote disease progression and recurrence. Such additional factors could be related to the response of the tumor cells to growth factors that are found at the tumor microenvironment, or to hormonal stimulation.

**Table 3:** TNFα and IL-1β prevalence in tumors of breast cancer patients

Cytokine	**Group No**.	Group name	% Positive cells in tumors (X ± SD)	Score (X ± SD)
**TNFα**	I	DCIS	67.3 ± 24.7	134.8 ± 82.4
	II	IDC-no-relapse	65.9 ± 23.7	141.9 ± 81.1
	III	IDC-with-relapse	85.0 ± 6.8	191.0 ± 70.3
				
		p value	0.0008	0.0331

**IL-1β**	I	DCIS	69.3 ± 21.6	117.6 ± 80.1
	II	IDC-no-relapse	55.7 ± 29.3	121.2 ± 96.5
	III	IDC-with-relapse	84.8 ± 11.5	206.2 ± 76.2
				
		p value	<0.0001	0.0004

The table provides information on the prevalence of TNFα and IL-1β expression in tumors of breast cancer patients, with analyses that were performed only on patients whose tumors expressed the cytokines: **DCIS group = Group I**: TNFα n = 15, IL-1β n = 18; **IDC-no-relapse group = Group II**: TNFα n = 17, IL-1β n = 15; **IDC-with-relapse group = Group III**: TNFα n = 29, IL-1β n = 31. Each of the TNFα- and IL-1β-positive tumors were analyzed for % positive cells in the tumors, intensity of expression of the cytokines, and score (as described in "Materials and methods"). The table provides the X ± SD of % cells positive for TNFα and IL-1β, and the X ± SD of the expression scores of TNFα and IL-1β in the three groups of patients.

For each cytokine, group means were compared by one-way analysis of variance. Whenever a significant effect of group was observed, pair-wise comparisons between groups were performed. The results of the statistical analyses have shown that: (1) For % TNFα-positive cells, p = 0.0008: Group III was statistically different from Groups I and II. (2) For TNFα score, p = 0.0331: Paired comparisons did not identify specific significant differences between the groups of patients. (3) For % IL-1β positive cells, p < 0.0001: All three groups were statistically different from each other. (4) For IL-1β score, p = 0.0004: Group III was statistically different from Groups I and II.

Supporting this possibility are analyses that we have performed, using the data obtained on the expression of Her2-neu, ER and PR in the patients. In this analysis, we have compared between the IDC-no-relapse and the IDC-with-relapse groups. Statistical analysis that was performed for IL-1β, taking into account the status of Her2-neu, ER and PR, all combined (currently each of these three factors is used in the clinic as predictors of disease progression and recurrence in breast cancer), indicated that IL-1β was a significant risk factor for disease relapse: p = 0.0402, with 95% confidence limits of 1.098 and 59.93 and odds ratio 1 value of 8.112 (also, of the different clinical markers, PR was statistically different between the IDC-no-relapse and IDC-with-relapse groups, with p = 0.0391). Similar analyses performed for TNFα, CCL2 and CCL5 did not reveal significant positive correlation with disease progression.

The findings on IL-1β strongly support the possibility that when the tumor-derived and/or microenvironment-derived characteristics have higher cancer-promoting properties, IL-1β can further promote progression-related processes in breast cancer.

## Discussion

In this study, we have identified the relationships between inflammatory chemokines and cytokines in breast cancer along different stages of disease, and have analyzed the potential roles of TNFα and IL-1β in promoting breast cancer progression. The results of this study shed light on the inflammatory setup in breast cancer, and provide three novel findings:

### (1) The expression of the inflammatory chemokines CCL2 & CCL5 and of the inflammatory cytokines TNFα & IL-1β is coordinated in breast cancer

To date, the relationships between inflammatory chemokines and inflammatory cytokines in breast cancer were not elucidated, and the associations between them during the process of breast cancer development and progression were not analyzed. At this setting, the findings of our study are important because they indicate that the expression of the inflammatory chemokines CCL2 & CCL5 and of the inflammatory cytokines TNFα & IL-1β is coordinated in crucial stages along the process of disease progression. Moreover, the expression of all four inflammatory factors is minimal in normal breast epithelial cells, and is simultaneously acquired by the cells once malignant transformation has taken place, namely from the DCIS stage and on. These results suggest that events dictated by genetic/epigenetic alterations in the tumor cells, or by the microenvironment, lead to a synchronized up-regulation in the expression of several inflammatory mediators together, by transformed breast epithelial cells.

The coordinated expression of CCL2 & CCL5 and of TNFα & IL-1β along stages of breast cancer development and progression is important because the activities of the four factors are not fully overlapping (Refs [3,5-24] for CCL2 & CCL5, [25-59] for TNFα and IL-1β). Therefore, it is possible that each of these factors contributes its own share to disease course, alongside with the others. Together, the coordinated presence of CCL2 & CCL5 and of TNFα & IL-1β may support malignancy, possibly also due to spatio-temporal cross-interactions between them. Indeed, our findings (Additional Files 1 and 2) and published studies [65-70] indicate that TNFα and IL-1β up-regulate the release of CCL2 and CCL5 by breast tumor cells. Furthermore, recent findings obtained in our studies suggest that TNFα and IL-1β exert tumor-promoting activities that are connected to the ability of CCL2 and CCL5 to function as cancer-supporting factors. Consequently, it is possible that the pro-tumorigenic activities of the inflammatory cytokines and of the inflammatory chemokines depend on each other, and/or are complementary to one another. This possibility emphasizes the need to determine whether combined inhibition of all factors together will lead to improved limitation of tumor growth.

Moreover, since our findings indicate that the coordinated array of cytokines and chemokines takes action in about 50-70% of the patients (depending on the group), it is possible that these factors can act along with other inflammatory and tumor-supporting factors to promote tumor growth and metastasis, as further discussed below.

### (2) The expression of TNFα & IL-1β is further increased in the IDC-with-relapse group of patients

In the group of IDC-with-relapse patients, the incidence of CCL2 & CCL5 expression was similarly high to that of DCIS and IDC-no-relapse group (50-65%), while that of TNFα & IL-1β has reached about 85-90%. Therefore, while the expression of CCL2 & CCL5 was coordinated with TNFα & IL-1β in many of the patients belonging to this group, there was a sub-population of patients in which the expression of TNFα & IL-1β was not accompanied by CCL2 & CCL5. While we need to understand the reasons for this lack of coordination in this specific sub-group of patients (please see discussion below), this observation indicates that TNFα & IL-1β are required, and play important roles in disease progression, and suggests that they cooperate with other factors at the tumor microenvironment that substitute for the lack of CCL2 & CCL5.

Of interest in this respect are the reasons for the lack of expression of CCL2 & CCL5 in the specific sub-population of IDC-with-relapse patients who do express TNFα & IL-1β. This lack of coordination between CCL2 & CCL5 and TNFα & IL-1β in this sub-population is intriguing mainly because TNFα and IL-1β were found to stimulate the release of CCL2 and CCL5 by breast tumor cells. The explanation could be given, at least partly, by consideration of the extent to which receptors for TNFα and IL-1β are expressed by the tumor cells, and of which type. Although the expression of TNFα & IL-1β receptors by breast tumor cells was already documented [29,31,37,43,44], it is possible that in this specific sub-group of the IDC-with-recurrence patients, the tumor cells do not express the required receptors for TNFα & IL-1β, or express non-signaling receptors. Under such conditions, TNFα & IL-1β would not act on the tumor cells to promote the release of CCL2 & CCL5, leading to lack of associations between the two cytokines and CCL2 & CCL5.

Accordingly, in our future investigations we will perform detailed analysis of the expression of receptors for the two cytokines (e.g. TNFRI, TNFRII, IL-1RI) in the course of disease progression in breast cancer. Such an analysis may enable us to better identify the basis for the patterns of expression of the inflammatory cytokines and chemokines in different stages of disease.

### (3) Tumors of IDC-with-relapse patients express persistent and high levels of TNFα & IL-1β, which may contribute to disease recurrence and progression, e.g. *via *EMT

As indicated above, the incidence of TNFα & IL-1β was further increased in IDC patients in whom disease has relapsed, suggesting that these two cytokines push forward processes of tumor progression and recurrence. Our findings support this possibility because they indicate that TNFα, and to a lower extent also IL-1β, induce EMT properties in the tumor cells.

In addition, our findings demonstrate that in order to undergo EMT, the tumor cells had to be constantly stimulated by TNFα. These results suggest that *in situ*, EMT processes would be facilitated by high persistence of TNFα at the tumor site. Under such conditions, we would expect high prevalence of TNFα in tumors of patients belonging to the IDC-with-relapse group, compared to IDC-no-relapse or to DCIS patients. Indeed, here we have obtained an important indication to the roles of TNFα in disease recurrence and progression, because there was significantly higher prevalence of TNFα in tumors of the IDC-with-relapse group than in the other two groups of cancer patients. In addition, significantly elevated persistence of IL-1β was also observed in the IDC-with-relapse patients. Although IL-1β was not a strong stimulant of EMT, it is possible that this cytokine supports tumor recurrence or metastasis by other means, such as increased angiogenesis, as indicated by several published studies [28,31,83], or by having joint activity with other factors. Supporting this possibility is the finding that in a specific setting of Her2-neu, ER and PR expression in the tumors, IL-1β was identified as a risk factor for disease recurrence, suggesting that it can act jointly with other pro-malignancy factors to promote disease progression in breast cancer.

The observations that were obtained in this part of the study support the possibility that high prevalence of TNFα and IL-1β expression in the IDC-with-relapse patients contributes to re-growth of the tumors and to metastasis formation by inducing processes that push forward these processes, e.g. *via *EMT and/or cooperative activity with other pro-malignancy factors that are expressed by the tumor cells, or by cells of the tumor microenvironment.

## Conclusions

Our study is the first to provide detailed analysis of the expression of inflammatory chemokines, alongside with inflammatory cytokines, in different progression stages of breast cancer. We provide evidence to a coordinated expression of the inflammatory chemokines CCL2 & CCL5 and the inflammatory cytokines TNFα & IL-1β in breast cancer, all having causative roles as tumor-promoting factors in this disease.

Our study also emphasizes the importance of TNFα and IL-1β in promoting disease metastasis and recurrence, gaining support by their high prevalence in patients in whom disease has relapsed. Such advantages could be given to the tumor cells by the ability of TNFα to promote EMT, and by similar (although to a lower extent) or other tumor-promoting activities of IL-1β (e.g. joint activities with other pro-malignancy elements). Mainly, the findings on TNFα contribute to the overall view of the roles played by this cytokine in breast cancer. While TNFα was found to have multiple tumor-promoting roles in this disease [25-59,84], there are malignant diseases in which it was described as an anti-malignancy factor [25,60,61]. Our present results provide further evidence to the major importance of TNFα in supporting breast malignancy, and strengthen the need to consider this cytokine as a therapeutic target in this disease.

Based on our findings, the possibility exists that different inflammatory mediators act in complementary manners at the tumor microenvironment to support processes of tumor growth and progression, with potentially major roles for TNFα. Such a possibility emphasizes the need to perform extensive in vitro and in vivo studies, in which the combined roles of such factors will be investigated, to be then followed by determining the effects of joint inhibitory measures on disease course. Indeed, in our analyses we are now taking the first steps in this direction. It is possible that such an approach may hamper different pro-tumorigenic mechanisms and signaling pathways simultaneously, leading to more inhibitory effects on breast tumor growth and metastasis.

## Abbreviations

DCIS: Ductal Carcinoma *In Situ*; EMT: Epithelial-to-Mesenchymal Transition; ER: Estrogen Receptor α; IDC: Invasive Ductal carcinoma; IHC: Immunohistochemistry. IL-1β: Interleukin 1β; PR: Progesterone Receptor; TNFα: Tumor Necrosis Factor α.

## Competing interests

The authors declare that they have no competing interests.

## Authors' contributions

GS was the major contributor to the study of normal individuals and breast cancer patients. She performed a large amount of the IHC stainings and was in charge of organization of the data, and also took charge of the migration and invasion assays. MOS was the major contributor to the EMT part of the project, and also performed some of the IHC stainings. IH coordinated the study in the Meir Medical Center, and performed some of the IHC stainings. NYH was involved in the initial stages of study design, and coordinated the study in Sourasky Medical Center. LLT was the pathologist who analyzed the biopsy sections. ES performed the statistical analyses. TLR has contributed to the migration and invasion assays. PW performed analyses related to EMT in the tumor cells. TM participated in EMT analyses. MG participated in the design of the study in Meir Medical Center. ABB was the principal investigator responsible for the whole study, including all its parts. All authors have approved the submission of the manuscript.

## Pre-publication history

The pre-publication history for this paper can be accessed here:

http://www.biomedcentral.com/1471-2407/11/130/prepub

## Supplementary Material

Additional file 1 **TNFa and IL-1b up-regulate the release of CCL2 by human breast tumor cells**. T47D (A) and MCF-7 (B) human breast tumor cells were stimulated for 24-48 hr with human TNFa or IL-1b. CCL2 levels were determined in the cell supernatants by ELISA, at the linear range of absorbance. The results are representatives of n ≥ 3. *p < 0.05, **p < 0.01 in comparison to unstimulated cells. Click here for file

Additional file 2 **TNFa and IL-1b up-regulate the release of CCL5 by human breast tumor cells**. T47D (A) and MCF-7 (B) human breast tumor cells were stimulated for 24-48 hr with human TNFa or IL-1b. CCL5 levels were determined in the cell supernatants by ELISA, at the linear range of absorbance. The results are representatives of n ≥ 3. **p < 0.01, ***p < 0.001 in comparison to unstimulated cells. Click here for file
