# Supplementary material for: Inflammatory mediators in breast cancer: Coordinated expression of TNFα & IL-1β with CCL2 & CCL5 and effects on epithelial-to-mesenchymal transition
Source: BMC Cancer. 2011 Apr 12;11:130. doi: 10.1186/1471-2407-11-130 (PMC3095565; doi:10.1186/1471-2407-11-130)
Supplement: Additional file 2 — TNFa and IL-1b up-regulate the release of CCL5 by human breast tumor cells. T47D (A) and MCF-7 (B) human breast tumor cells were stimulated for 24-48 hr with human TNFa or IL-1b. CCL5 levels were determined in the cell supernatants by ELISA, at the linear range of absorbance. The results are representatives of n ≥ 3. **p < 0.01, ***p < 0.001 in comparison to unstimulated cells. [file 1471-2407-11-130-S2.PPT]

## Slide 1
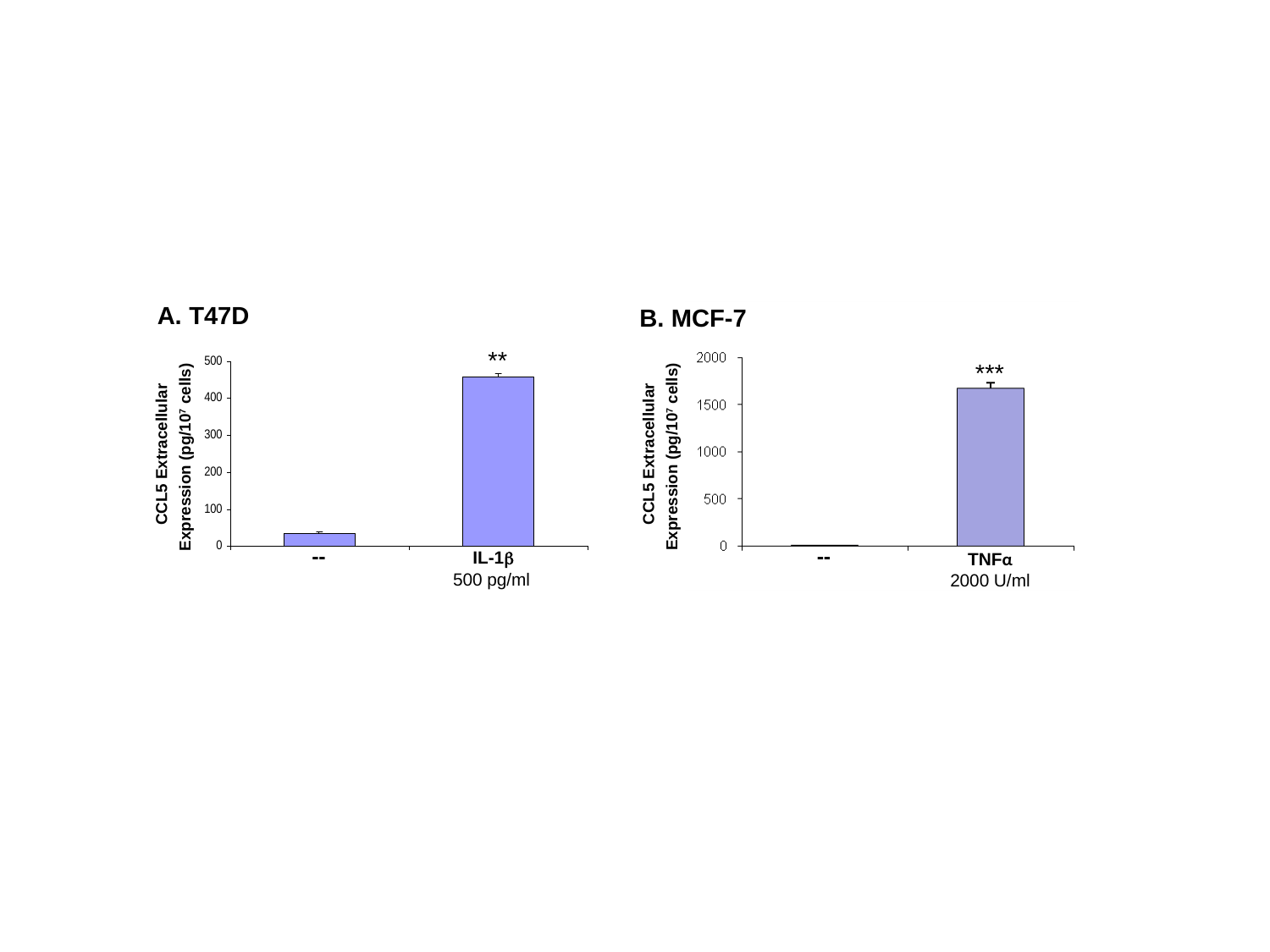

A. T47D
B. MCF-7
**
***
CCL5 Extracellular
Expression (pg/107 cells)
CCL5 Extracellular
Expression (pg/107 cells)
--
-- IL-1β
 500 pg/ml
TNFα
2000 U/ml
